# 

**Published:** 1833-02

**Authors:** 


### MONTHLY LIST OF MEDICAL BOOKS.

[*Medical Works cannot be entered on this List unless a copy be sent for the purpose, the titles of Books having frequently been sent to us as published, which have not appeared for weeks, or even months, after.*]

The Cyclopædia of Practical Medicine. Edited by John Forbes, M.D., F.R.S., &c. Alex. Tweedie, M.D., &c., and John Conolly, M.D., &c. Part XIII. (published in Monthly Parts,) January 1833. Containing *Inflammation* (continued), by Dr Adair Craufurd and Dr. Tweedie; *Influenza,* Dr. Brown; *Insanity,* Dr. Prichard; *Irritation,* Dr. C. J. B. Williams; *Ischuria Renalis,* Dr. Carter.—Large 8vo. pp. 115. Sherwood, Gilbert, and Piper; and Baldwin and Cradock, London.

On the Structure of the Human Placenta, and its Connexion with the Uterus, &c. By Thomas Radford, senior Surgeon to the Lying-in Hospital and the Dispensary for Diseases of Women and Children, and Lecturer on Midwifery.—8vo. pp. 23; three plates. Manchester.

A Further Examination of the Principles of the Treatment of Gout, with Observations on the Use and Abuse of Colchicum. By Sir Charles Scudamore, M.D. F.R.S. &c. Second Edition, considerably altered and enlarged.—8vo. pp. 127. Longman and Co.

### APOTHECARIES' HALL.

NAMES OF GENTLEMEN to each of whom the Court of Examiners have granted Certificates:

JAN. 3.

John Crouch, of St. Croix, Hants.
Thomas Dowse, Skelmanthorpe
Henry Dowland Haskins, Oxford
James Hancock, Hawarden
Thomas Ward, Tonbridge.

JAN. 9.

Joseph Baines, Bradford, Wilts.
Francis Fullager, Chichester
Thomas Norris, Hull
Wm. Charles Stedman, Portsmouth.

JAN. 17.

Samuel Ford Allnutt, Portsea
John Morgan Bryan, Northampton
John Wm. Cartledge, Chesterfield
Christopher Elliott, Pilltown, Ireland
James Flesher, Tiffield, Northamptonshire
Uniacke Ranayne, Lismore.

JAN. 24.

Pye Henry Chavasse, Birmingham
Henry Hannam Cort, Ripley, Surrey
Thomas Young Cotter, Lisson Green
Henry Pye Lewis Drew, Gower street
John Evans, Farningham
Samuel Edward Fitch, Cambridge
Richard Brinsley Hinds, Brunswick square
Joseph Hutchinson, Lincoln
Edward Parke, Liverpool
Edward Williams Richards, Tregaron
Wm. Waring Saxton, Whitchurch, Salop
Moses Thompson, Pontefract
Wm. Wilton, Guildford.

---

